# Twenty Years of “Lipid World”: A Fertile Partnership with David Deamer

**DOI:** 10.3390/life9040077

**Published:** 2019-09-20

**Authors:** Doron Lancet, Daniel Segrè, Amit Kahana

**Affiliations:** 1 Department of Molecular Genetics, Weizmann Institute of Science, Rehovot 7610010, Israel; amit.kahana@weizmann.ac.il; 2 Bioinformatics Program, Department of Biology, Department of Biomedical Engineering, Boston University, Boston, MA 02215, USA

**Keywords:** Lipid World, origin of life, GARD, micelle, systems chemistry, systems protobiology, compositional inheritance

## Abstract

“The Lipid World” was published in 2001, stemming from a highly effective collaboration with David Deamer during a sabbatical year 20 years ago at the Weizmann Institute of Science in Israel. The present review paper highlights the benefits of this scientific interaction and assesses the impact of the lipid world paper on the present understanding of the possible roles of amphiphiles and their assemblies in the origin of life. The lipid world is defined as a putative stage in the progression towards life’s origin, during which diverse amphiphiles or other spontaneously aggregating small molecules could have concurrently played multiple key roles, including compartment formation, the appearance of mutually catalytic networks, molecular information processing, and the rise of collective self-reproduction and compositional inheritance. This review brings back into a broader perspective some key points originally made in the lipid world paper, stressing the distinction between the widely accepted role of lipids in forming compartments and their expanded capacities as delineated above. In the light of recent advancements, we discussed the topical relevance of the lipid worldview as an alternative to broadly accepted scenarios, and the need for further experimental and computer-based validation of the feasibility and implications of the individual attributes of this point of view. Finally, we point to possible avenues for exploring transition paths from small molecule-based noncovalent structures to more complex biopolymer-containing proto-cellular systems.

## 1. Carving a Fertile Collaboration

In November 1999, a paper entitled “The Lipid World” [1] was submitted to the journal Origin of Life and Evolution of the Biosphere (OLEB). Three of the paper’s authors were Doron Lancet and two of his graduate students at that time, Daniel Segrè and Dafna Ben-Eli from the Weizmann Institute of Science in Israel. The fourth author was David Deamer from the University of California Santa Cruz, USA.

Doron Lancet recalls some of the steps that led to the collaboration with Dave Deamer, which gave birth to Lipid World: In 1998, Lancet was a relative novice to the field of the Origin of Life. Until 1994, his research focused on immunity, olfaction and genomics [2,3,4,5]. This included a paper entitled “Probability Model for Molecular Recognition in Biological Receptor Repertoires”, deriving a formula that underlies multi-receptor repertoires in immunity and olfaction [6]. Upon interaction with Origin of Life pioneers in Israel, such as Shneior Lifson [7] and Noam Lahav [8], Lancet realized that his probability model for molecular recognition could generate new insight on life’s origin. This led to research conducted with his graduate student Yitzhak Pilpel (presently professor at the Weizmann Institute), and Ora Kedem, a world luminary in irreversible thermodynamics [9]. Using an analog of the Receptor Affinity Distribution applied to catalysis, we were able to demonstrate the emergence of order and reproduction behavior in autocatalytic sets of oligomers [10], in the footsteps of Stuart Kauffman’s autocatalytic sets [11]. With Daniel Segrè, we later showed that the specific shape of this affinity distribution is in fact an essential ingredient for the emergence and maintenance of this homeostatic behavior [12].

In 1998, Doron Lancet got a letter from David Deamer asking if he could visit his research group for a sabbatical. By that time, Deamer had published dozens of papers on prebiotic evolution, so Lancet felt honored and elated, immediately inviting him to come. In the preceding couple of years, Daniel Segrè, a particle physicist by training and now Professor at Boston University, still publishing on life’s origin [13,14], began working with Lancet as a Ph.D. student. Together, we extended the laboratory’s earlier prebiotic foray, and better established the Graded Autocatalysis Replication Domain (GARD) model, which we used to perform computer simulations of the origin of reproduction and inheritance in non-equilibrium mutually catalytic networks. Lancet recalls that having such a specific framework made him more secure in his capacity to be an appropriate sabbatical host for Deamer.

What made Lancet and Segrè even more excited was that in his prior publications, Deamer carved a significant niche, claiming a central role for lipids in life’s emergence [15,16,17,18,19]. This facet was especially enticing because in the previous couple of years, inspired also by the work of Pier Luigi Luisi [20], they had started exploring the idea of mutually catalytic networks whose main molecular constituents are amphiphilic molecules. We realized that the coupling of mutual catalysis with spontaneous aggregation into micelles and vesicles provided a compelling putative path from prebiotic chemistry to the first self-reproducing and evolving systems [21]. Two years later, this foundation led to the full-fledged mathematical formulation of a variant of the GARD model in which catalysis of the aggregation process itself could give rise to supramolecular structures capable of growing and dividing. Inspired by calculations of protocell inheritance statistics previously proposed by Harold Morowitz [22], we discovered that our simulations could give rise to the nontrivial inheritance of metastable compositions. In analogy with genomes, we named these inheritable dynamic supramolecular structures compositional genomes, or “composomes” [23]. We further deepened our confidence in the relevance of lipid molecules as possible players by finding published evidence for extensive and diverse catalytic capabilities of amphiphilic molecules within micelles ad vesicles [24] and by showing that their documented catalytic parameters conformed to the probability distribution we had hypothesized to be crucial (see [1] chapter 7). Our model, taking into account biochemically inspired statistical properties of kinetic and thermodynamic parameters, provided for the first time in silico evidence that lipid mixed assemblies that grow and split can transmit molecular information from one generation to another. This suggested that replicating RNA polymers were not the only option for life inception.

## 2. Double-Faced Lipid World

Thus, when Dave Deamer arrived at the Weizmann Institute in 1998, we decided to write a paper together on the different roles lipids could play in life’s origin. We felt that it would be revealing to join Deamer’s broad vista on the experimental evidence for such view with our just-emerged formal perspective on novel roles for lipids. Thus, the Lipid World paper [1] represented an amalgamation of two complementary lines of thought. The first constituted carefully accumulated experimental data suggesting that amphiphiles were prebiotically abundant, and that amphiphile-made compartments likely played a key role in protocell emergence [25,26]. The second was an enterprising use of physicochemical principles to suggest what many considered an improbable premise: that beyond forming compartments, lipid assemblies (or, truly, any molecules capable of transiently and dynamically self-aggregate) could in principle store, copy and transmit information.

It is noteworthy that even the conservative view of the role of lipids in life’s origin is still debated, as summarized [27]: “there are…plausible origin of life scenarios that do not require the formation of an explicit boundary, at least not initially”, with support from publications on hydrothermal vents [28] and Iron-Sulfur World [29]. But by and large, with much credit to Dave Deamer, the central role of compartments in life’s origin is widely recognized. This was echoed in a recent review [30] and an impressive new book [31] by Deamer.

Deamer’s review opens with the sentence, “At some point in early evolution, life became cellular”, suggesting that lipid compartments were indispensable, but not necessarily first. How lipids could have joined forces with RNA is articulately described in the book (page 138), mirroring a widely accepted dogma: “…life can begin with chance ensembles of encapsulated polymers, some ... store genetic information in the linear sequences of their monomers while others catalyze polymerization reactions”. RNA is thus illustrated as the key player, having been the first to emerge, and subsequently joining forces with the compartment-forming lipids. A very similar viewpoint is expressed in a review by Koonin and co-workers on the evolution of primordial membranes: “(our analyses) predicated on the standard model…(the) assumption that the emergence of RNA and proteins preceded the appearance of membrane-encased life forms” [32]. But in the same article, the authors also mention “alternative models are available, including origin of life in a ‘lipid world’”.

Indeed, the second facet of the Lipid World paper delineates an origin path that begins with lipids, or—more broadly—any type of amphiphilic molecule. In conjunction with the preliminary outlook provided by the early GARD simulations, this paper described in detail published evidence for catalytic properties of lipids and their aggregates. In fact, to highlight the fact that catalysis is not a property reserved to proteins or RNA, we coined the term “lipozyme” to label non-covalent amphiphilic aggregates endowed with catalytic capacities. The same section further highlighted the likelihood that early amphiphiles were highly diverse, a prerequisite for compositional diversity. In addition, it summarizes previously computer-derived evidence that autocatalytic and mutually catalytic lipozymes could make their own copies upon growth and fission. The upshot is the ensuing hypothesis that lipid assemblies could replace RNA as the first chemical entity capable of reproduction. Calling our paper “The Lipid World” was aimed to imply such possible alternative to “RNA World”. This juxtaposition highlights the concrete possibility that self-reproduction could have been achieved through mechanisms that are simpler than template duplication of sequences, but does not rule out the possibility that lipid-based ensembles and nucleotide precursors or biopolymers may have co-occurred and coevolved at some stage of life’s origin.

One of the forerunners of a similar claim was Alec Bangham, who was the first to report, in the 1960s, the formation of artificial liposomes [33]. He was so impressed with the spontaneity in which lipid membrane structures formed and by their life-like behavior growing, changing shapes, splitting and passing solutes, that he claimed that “Membranes came first”. This formative moment in the research history of prebiotic lipids is described by Deamer in a memoire [34] and illustrated vividly in another paper in this special issue [35]. The collaborative Lipid World paper critically examines Bangham’s pioneering hunch.

What has been done in this respect, and a possible roadmap for the future, is detailed in a recent retrospective review by Lancet’s group [36]. Among other topics, the review advocates a systems protobiology viewpoint, whereby the first replicators (or more accurately reproducers [37]), were “assemblies of spontaneously accreting, heterogeneous and mostly non-canonical amphiphiles”. This proclamation hides several dissenting claims on life’s origin: First, life could have begun with chemistries quite different from those of present-day life, taking advantage of what was randomly available, rather than relying on very specific abiogenic syntheses instructed by present-day life. Notably, however, recent work by Segrè and colleagues [13,38] has explored possible trajectories for the evolution of biochemistry, suggesting that metabolism today still carries information on its very early history, and showing that a variety of biomolecules, including rudimentary lipids, could have been sustainably produced by a pre-enzymatic metabolism. Second, early life might have strongly depended on molecules that could spontaneously accrete into non-covalent ensembles of mutually interacting compounds (systems from inception). Third, in the RNA case, the constitutive monomers are strung together by extraneous catalysts and self-copied via a very specific base-pairing path. In contrast, in the lipid case non-covalent “stringing” occurs spontaneously, and reproduction stems from an endogenous catalytic network with diverse mutual catalytic events deriving from nature’s probability distribution, thus resembling a primitive metabolism [39].

The circumstance of Lipid World spelling an alternative to RNA-first should allow scientists to judge the pros and cons of two competing scenarios for the early rise of self-reproduction, in the spirit of the scientific method, as exemplified [40]. This process has begun during Dave Deamer’s Sabbatical with us. A fertile discussion ensued at that time: Deamer warned that it may be much too risky to go as far as invoking information-copying in lipid assemblies without experimental evidence. Lancet’s stand inclined towards the notion that a hypothetical framework, which leads to a model maximally conforming to the law of organic and physical chemistry, is a legitimate roadmap for future chemical and astrobiological observations and experiments. Segrè, while fond of the power of mathematical and computational approaches, became convinced of the importance of experimentally showing proof-of-principle feasibility of compositional inheritance. Our joint paper attests to a productive mutual acceptance of these disparate points of view.

**Figure 1:**
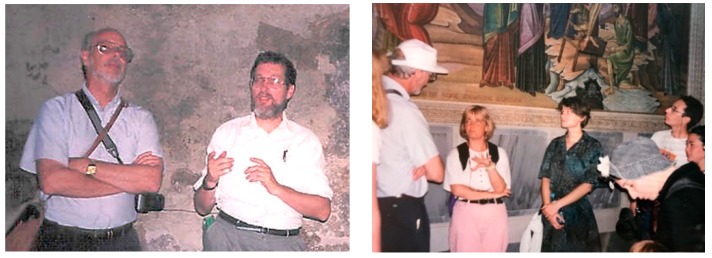
Deamer (left on both figures), Segrè (right end on right figure) and Lancet (right side on left figure) touring Israel, 1998.

**Figure 2:**
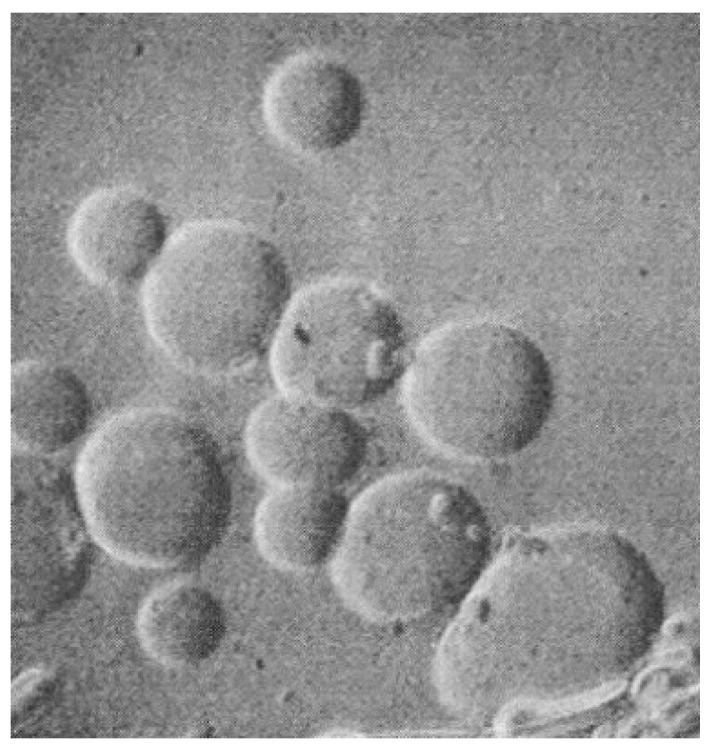
Figure from Segrè’s notebook documenting liposome formation during Deamer’s sabbatical.

An amusing relevant recollection from Deamer’s visit is that he and Daniel Segrè devoted a couple of months at Weizmann to preliminary laboratory experiments attempting to design and observe autocatalytic liposome growth. For Dave’s 80^th^ birthday, the three of us fondly shared memories (Figure 1) as well as Daniel’s surviving laboratory notebook documenting his experiments (Figure 2).

Twenty years later, experimental verification is still hard to attain. One possible reason for this is that network-based evolutionary progression likely stretched over hundreds of millions of years (cf. [36] chapter 13) and is difficult to recapitulate in standard laboratory conditions. It is possible that under appropriate experimental settings, this process could be recapitulated in a much shorter time. Still, a typical such experiment would require following the detailed chemical composition of a large number of noncovalent structures under nonequilibrium conditions over long periods of time, which at present, is definitely challenging. Therefore, as for deciphering stellar and galactic evolution, it might be necessary to use computer simulation, including Molecular Dynamics, for arbitrating different life origin scenarios [41]. Another possible reason for the lack of experimental verification is an overall hesitancy in the acceptance of the paradigm-shifting concept of compositional inheritance, with the consequence that a relatively small (though growing) number of laboratories have embarked into projects that could contribute to testing its validity.

## 3. Twenty Years of Lipid World

The perspective afforded by the passage of time since 1998 allows us to ask how far science has in fact, gotten in such arbitration, and how that encounter and the resulting paper have been weaved into origin of life research and conversations. Scrutinizing the >400 Google Scholar citations for “The Lipid World”, we realized that a majority of them cite this paper just to indicate the possible existence of a lipid-first scenario in parallel to others such as RNA first, protein first, carbohydrates first, metabolism first and pyrite first. Many of these citations do not address, support or negate, the “unorthodoxy” of lipid-based catalysis and lipid-based reproduction.

A noteworthy type of citations are those highlighting the fact that “vesicles…could have undergone…a kind of self-reproduction, in the sense that new vesicles are produced by growth and dispersion of preformed vesicles” ([42] p. 206). Such growth-split phenomenology, a visual mimic of the proliferation of living cells, has limited relevance for the unorthodox Lipid World. This is because just growth and fission are necessary conditions for bona-fide lipid reproduction, but not a sufficient condition. Reproduction in its true meaning requires that specific information be preserved along the growth-fission process, and this can only happen if the original assembly has compositional information based on the heterogeneity of its constituents. It is the ensuing non-trivial concentration-preserving (homeostatic) growth, followed by random fission, that marks true lipid reproduction, as descried in the Lipid World paper ([1] chapter 9) and further elaborated in numerous publications since [23,36,43].

The Lipid World paper brings up a highly important point regarding compositional homeostasis and homeostatic growth, as follows: “It is important to point out that when present-day cells divide, they too transmit considerable elements of compositional information (including specific gamuts of lipids, proteins and RNA) which are ‘inherited’ from the mother cell”. This alludes to the analogy between protobiotic compositional inheritance in lipid assemblies and epigenetic inheritance that plays a key role in present-day life, as further expounded [36].

Indeed, several publications that cite the Lipid World paper focus on epigenetics. One prominent example is a paper [44] pointing out that in addition to DNA, reproduction and development require epigenetic factors that cannot be reduced to genetics. Importantly, the authors stressed that such “epigenetic factors can be traced back to the origins or very early evolution of life.” They claimed that among life origin scenarios, RNA World is the least compatible with epigenetics, and that it is popular “because it fits the gene-center line of thought, according to which genetic factors are the most fundamental” and everything else is a derivative. The authors emphasize that there are other origin scenarios that are fully compatible with the centrality of epigenetics in contemporary life—metabolism and cell first, including the Lipid World—GARD composomes scenario.

The analogy between reproduction as delineated in the Lipid World, and epigenetic inheritance goes even further in the case of modern cellular organelles which, as described by Cavalier-Smith under the title “genetic membranes”, involve membranes that “always arises by growth and division from preexisting membranes of the same kind (e.g., chloroplast envelope). In effect, genetic membranes constitute the germline of membrane heredity” [45]. Such insight strengthens the position of lipid inheritance, governed by a network of mutual rate enhancement events. In fact, in full analogy, the lipid compositional homeostasis of present-day organellar membranes (each organelle type having its idiosyncratic composition) is mediated by specific catalytic transporters, part of the modern metabolic network [46].

Another article that cites the Lipid World paper in its unorthodox incarnation constitutes one of the most systematic renderings of the limitations of the RNA World view [47]. Employing phylogenomics, which combines genome data and evolutionary reconstructions, they “retrieve deep historical signals from a census of molecular structures and functions in thousands of nucleic acid and protein structures and hundreds of genomes”. They conclude: “Our findings falsify the existence of an ancient RNA world; instead (our results) are compatible with gradually coevolving nucleic acids and proteins in interaction with increasingly complex cofactors, lipid membrane structures and other cellular components.” This foregoing statement attests to capacity of the scientific method to empirically arbitrate between the different origin scenarios and serves as a reminder that one should not let the labels of these scenarios set sharp artificial boundaries between different chemistries that likely had a complex co-evolutionary history.

Further stating that “modern biochemistry is the result of the gradual evolutionary appearance and accretion of molecular parts and molecules”, the authors highlight just one alternative to RNA, the GARD model, in the context of the ‘lipid world’ scenario. They describe how, in this model, “information in the composition of amphiphilic molecules that interact with each other noncovalently enable growth in evolution of primordial container systems”. They stress that “this is done without the need of information that is internal to the individual components, i.e., sequence or structural information of the amphiphiles”. This portrayal continues by mentioning that GARD was technically contested in [48] due to poor evolvability, but that a later paper from our group [49] negated this doubt by showing that GARD harboring mutual catalysis rather than self-catalysis was more robust and responsive to selection. The authors conclude that “GARD has the potential to explain the emergence of self-sustaining catalytic ensembles that benefit ‘metabolism-first’ or even ‘protein-first’ scenarios of origin without invoking a replicator/genetic component”.

Indeed, beyond the specific embodiment of the original GARD formulation, the possibility that purely compositional reproduction could be endowed with enough complexity to display a process akin to evolutionary adaptation, in which historical accidents can get fixated and built upon, remains an open question, and—we claim—one of the most profound questions in the origin of life. There is no fundamental reason for this process to be unfeasible. In fact, modelling of the original GARD ‘Lipid World’ shows encouraging indications of evolutionary capacities, beyond mere heredity. This includes responses to environmental changes and takeover events among competing compositional lipid networks, as reviewed [36]. If full-fledged evolution is finally shown to be possible, such a mechanism would provide the conceptually missing bridge between a pre-Darwinian abiotic planet and Darwinian evolution as we know it today. Whether through more advanced mathematical models, computer simulations or experimental techniques, we anticipate and wish that a lot more research will address this question in the future.

The same paper mentioned above [47] subsequently mentions a fault apparently shared between RNA World and Lipid World, saying that “despite its lure, the ‘worlds’ paradigm is utopian and nonproductive”. The reason is that both life today and the primordial chemistry are ‘messy’ [50,51] and that “It is highly unlikely that replicators and compositions of the primordial world were originally of a same kind and were less rather than more stochastic than the biological systems that exist today” ([47] p.155.). This criticism, on which we are in complete agreement, highlights the limitations of the RNA-first approach, because this scenario adheres to highly specific chemistries. It also highlights the limitation of the Lipid-first scenario, but at a very different level. While a narrow interpretation of the Lipid World hypothesis may indeed, similar to the RNA world, focus on the necessity of specific molecules (e.g., phospholipids) for early inheritance, the core concept of noncovalent assemblies as early reproducers is much broader, and not arguing at all for the need of components “of the same kind”. In fact, behind the term lipid, even in present-day life hides a vast chemical diversity [52]. The unconstrained prebiotic diversity can easily be orders of magnitude higher ([1] chapter 2) whereby practically any member of such “combinatorial library” can take part in supramolecular assembly dynamics, described as the model’s inherent promiscuity ([36] chapter 2). Notably, there is experimental evidence for the appearance of such diversity in planetary primordial organic matter [53].

We sometimes wonder whether the choice of Lipid World as the label of our work back then may have indeed been largely interpreted as claiming the primacy of some specific chemical structures over others, as opposed to the overall idea that noncovalent structures could have predated biopolymers. Still, especially in light of the way this work stemmed from our own immersion into the world of lipids during Deamer’s sabbatical, we look back with affection at that choice. For clarification, all along subsequent Lipid World research, we have stressed that the term “lipid” is used in its broadest context [36], including a diversity of aggregate-forming molecules, such as amphiphilic peptides [54,55], and apolar molecules in micelles and nanoemulsions [56].

Finally, the same paper [47] raises yet another shortcoming that affects Lipid World: how lipid assemblies could beget nucleic acids and proteins in interaction with metabolism. One possible answer, in addition to the obvious argument that the internal aqueous space of a vesicle can serve as a micro-bioreactor for chemical synthesis, is that under the “lipid” umbrella, there can be diverse life-like headgroup types, including amino acids and peptides, as well as mononucleotides and oligonucleotides ([36] chapter 11.2). In the strongly interactive environment of a lipid assembly, a fertile arena for coevolution emerges. In other words, while it is extremely difficult to see how RNA begets proteins and lipids, lipids with the right headgroups may give rise to proteins and RNA. This state of affairs, whereby GARD assumes mutually catalytic covalent oligomerization [43,57] and also portrays resemblance to cellular metabolism is described in ([36] chap. 11.1), under the title of Metabolic GARD or M-GARD.

In his recent book [31], chapter 10, David Deamer addresses the important question of early life’s adaptation to new niches. He writes “This stepwise adaptation will involve encapsulated polymer systems, but the membranous boundaries can also adapt different compositions that tend to stabilize them”. He mentions examples such as lipid bilayer stabilization by the nucleobase adenine or by polycyclic aromatic hydrocarbons, similar to the effect of cholesterol in modern membranes. Finally, he comments: “The Lipid World proposed by Segrè et al. (citing the Lipid World paper) hints at this kind of compositional evolution”. We note that static membrane stabilization by the inclusion of certain compounds is conceptually different from GARD’s composition-dependent dynamic preservation that lasts along many growth-split generations.

## 4. The Future of Lipid World

Significant leaps in future studies of the role of lipids in the origin of life, following in the footsteps of our original paper, will require novel experimental, theoretical and computer-based evidence. From a computational perspective, it will be important to continue the development of mathematical models of ensembles of mutually catalytic molecules. While simplified chemistries, like the one of the GARD model are useful for studying the rise of homeostasis and reproduction, there are plenty of opportunities for extensions to more realistic chemistries, and more detailed modeling approaches. At the same time, one should also not discard mathematical models due to their simplicity, as they may point to relevant general principles. In short, there is room for many different types of models, and the claim that a model is abstract and mathematical (cf [58]) does not imply that it cannot help advance the understanding of life’s origin.

A thorough testing of the GARD model is portrayed in a paper by Guttenberg et al. [59], which serves as a good example for future computational analyses. The authors developed a metric to compute the amount of heritable variation enabled by a dynamical system. The method determines the number of heritable sets in models with no clearly defined genomes. The authors selected the GARD model as a test ground, because it is “a non-trivial model system that has a distributed, compositional heredity, which has been extensively studied”. They further point out that the “Lipid World hypothesis (defines) cells that concretely identify the ‘individuals’ to which a given composition belongs”.

Guttenberg et al. report on GARD simulations they performed by the method previously described [49]. They obtained the count per simulation of composome types (compotypes), which are GARD’s heritable states, doing the simulation with different degrees of selection pressure. In parallel, they assess the heritable states counts by their own algorithm. The results showed adequate agreement between the two sets of results, attesting to the validity of the notion of lipid assembly inheritance. The two algorithms were also in agreement regarding how the number of inheritance states changed with molecular assembly size. Finally, the results appear contradictory to those of another study of the GARD model [48], which reported negligible inheritance in GARD. Guttenberg et al. explain this dichotomy as stemming from a difference between intrinsic variation around one fitness optimum, as opposed to variation between fitness optima.

One other concept that will require convincing future corroboration is that composition is a valid form of cross-generational information transfer. A highly relevant study by Paul Higgs [60] has investigated this question in depth. The study highlights the complexity of the genetic apparatus controlling inheritance in modern cells and brings forth the question of whether “some other form of heredity is possible without polymers”. The authors then bring forth the Lipid World scenario “which considers non-covalent assemblies of lipids…(that) store information in…non-random molecular composition, …passed on when the assemblies divide, i.e., show compositional inheritance”. To study the generality of this mechanism, the authors compare the kinetically based mutual catalysis conduit used by GARD to a thermodynamic model based on mutual interaction energies. Their results confirm that compositional inheritance occurs in both models, attesting to the validity and generality of the compositional inheritance concept.

One of the arduous future challenges will be obtaining experimental evidence for compositional inheritance in non-covalent assemblies. It is tempting to propose mixing dozens of lipid types together at some different combinations and hope to see homeostatic growth in action. But the “experiment” that took place on early earth leveraged the immense time periods and spatial expanses on the Hadean planet ([36] chapters 13). With the more modest resources available to experimentalists, the success of this endeavor seems improbable due to combinatorial complexity. What could come to the rescue is the demonstrated attractor properties of the evolution of life in chemical composition space [39,61], which might diminish the improbability hurdle. In parallel, Molecular Dynamics, which can precompute a composome for a set of realistic amphiphiles, could allow the experimenter to choose the correct molecular combinations [41].

If the validation of compositional reproduction in lipid assemblies has to be deferred, it will be highly advantageous to at least obtain evidence for the molecular properties needed for such ensemble behavior. An important component of this will be extending the experimental evidence for the catalytic capacities of individual amphiphiles and their assemblies, both for covalent and non-covalent reactions. Considerable such evidence has already been portrayed in the Lipid World paper (see Section 2 above), and more substantiation has been reviewed recently ([36] chapter 10.1). Curiously, despite appreciable long-available published evidence in this vein, a common criticism remains that lipids are incapable of catalysis. For example, Lazcano [62] states: “Although (they) conclude that reciprocal catalysis among prebiotic amphiphilic molecules could have led to vesicle growth and multiplication, their calculations seem to pay little attention to the chemical properties of the components of their lipid world”. Notably, a statement that lipids are absolutely unable to catalyze cannot be upheld, because the promiscuity of the polar lipid head groups should encompass the diverse documented collection of non-enzymatic catalysts [63]. Furthermore, the attachment of a hydrophobic tail to a soluble non-enzymatic catalyst has been shown to enhance its catalytic properties [64].

An equally important future development would be showing a believable path for the transition from a “lipid only” setting to what would be considered bona-fide protocells. For this, it would be crucial to envisage how the “pillars” of protocellular chemistry could emerge in a rudimentary evolutionary process, occurring within modest micelles composed only of lipid-like amphiphiles (see previous section). This should include the emergence of the following: (a) More sophisticated lipids assemblies, i.e., vesicles with aqueous lumen; (b) Increasingly long membrane-attached peptides and integral membrane proteins with improving catalytic and recognition power, as described [65]; (c) Simple oligonucleotides (membrane attached or luminal) that show a measure of base-pairing dynamics, and interact with peptides in ways embodied in very early modes of encoding, as proposed [66]; (d) Metabolites attached to the membrane and contained in the lumen, that feed the synthetic paths of items (a)–(c) and include auxiliary compounds such as cofactors with non-enzymatic catalytic properties. Significantly, such protocells would not need to be neither purely autotrophic nor purely heterotrophic. In other words, some of the protocellular constituents would be imported from the environment and others produced within the gradually evolving endogenous mutually catalytic network. Similar scenarios that do not include replicating polynucleotides as a prerequisite for life’s emergence have been drawn in considerable detail in papers [67,68] that cite Lipid World.

In an overview, the Lipid World focuses on heredity of self-assembling structures, whereas the essence of the complementary view of the RNA World hypothesis is the emergence of self-replicating biopolymers and the transit to another biopolymer via the encoded synthesis of proteins. More details on research on possible evolutionary processes which link the ‘lipid world’ to such modern components are depicted under the auspices of the Metabolic GARD model [36], as alluded to in the previous section and below.

Future work could continue exploring the abovementioned metabolic GARD model, to push its limits in the capacity to generate paths towards more elaborate protocells, which may gradually acquire templating biopolymers. This will require both a more detailed delineation of the model itself, as well as availability of estimates for key experimental parameters. A specific embodiment of such simulations could test the feasibility of homeostatic growth and reproduction of vesicles with novel lipids generated by lipozyme catalysis, as well as reproduction of the lumenal content [36]. These simulations should be accompanied by parallel laboratory evidence of individual processes relevant for such behavior, including recent evidence of mutually catalytic lipid networks [69,70,71], and amplification of chirality through self-replication of micellar amphiphile assemblies [72]. Future expansion of such evidence will help unfold the complexification of the Lipid World. Finally, in light of recent theoretical and experimental evidence for the feasibility of spontaneously arising nonenzymatic proto-metabolic networks resembling core metabolic pathways present in extant life [14,73,74,75,76], it will be interesting to explore links between these self-sustaining sources of key biomolecules and the emergence of lipid-based ensembles like those described in the Lipid World.

## 5. Concluding Remarks

The timely and exciting encounter of Deamer with our group resulted in the joint writing of the Lipid World paper. This turned out to be a highly cited compendium on why lipids and other amphiphiles must have been key players at different stages of life’s origin. In parallel, this joint paper became one of the earliest showgrounds for some innovative ideas regarding how noncovalent assemblies might have constituted early reproducers, potentially preceding the much more advanced system of replicating nucleic acid encoders.
